# Improving Surgical Outcomes in Resource-Limited Settings: A Quality Improvement Approach to Unplanned Returns to Theater

**DOI:** 10.7759/cureus.82732

**Published:** 2025-04-21

**Authors:** Sarah Al-Musawi, Kamal Al-Jawdah, Firas Mahmood Rashid Al-Musawi

**Affiliations:** 1 Obstetrics and Gynecology, University Hospitals of Northamptonshire, Northampton, GBR; 2 General Surgery, Lincolnshire Community and Hospitals National Health Service (NHS) Group, Lincolnshire, GBR; 3 General Surgery, Al-Kindy Teaching Hospital, Baghdad, IRQ

**Keywords:** gynecology and obstetrics, patient safety, post-surgical complications, quality improvement projects, resource-limited settings, return to theater, unplanned reoperation, who surgical safety checklist

## Abstract

Background: Unplanned return to theater (RTT) is a major concern in surgical care, often reflecting preventable complications that impact patient safety and health system efficiency. While quality improvement strategies are well-established in high-resource settings, their implementation in low-resource environments remains underexplored.

Objectives: This study aims to evaluate the impact of a multi-modal quality improvement program on RTT rates in general surgery and obstetrics and gynecology at two tertiary hospitals in Iraq.

Materials and methods: This two-center, quasi-experimental study included 1,133 surgical cases over two cycles: a retrospective baseline (January 2021-January 2022) and a prospective post-intervention phase (March 2022-March 2023). Interventions included staff training on secure surgical knot techniques, enhancing adherence to the WHO surgical safety checklist, and mandatory supervision by consultants during high-risk operations. Key outcomes included RTT rates, causes, preventability, intensive care unit admissions, and hospital stays.

Results: The overall RTT rate decreased significantly from 7.9% in the first cycle to 3.7% in the second (p = 0.004). Reductions were observed in both general surgery (6.3% to 3.0%, p = 0.048) and obstetrics and gynecology (10.5% to 4.8%, p = 0.037). Sepsis (40.0%), wound dehiscence (34.3%), and hemorrhage (25.7%) were the leading causes of RTTs. Incomplete WHO checklist documentation and urgent surgeries were significantly associated with RTTs (p < 0.001). Most RTT cases (58.6%) were deemed non-preventable, but 32.9% were potentially preventable, and 8.5% were preventable.

Conclusions: A structured, low-cost quality improvement program effectively reduced RTT rates in two Iraqi hospitals. Interventions such as surgical technique training, checklist compliance, and consultant supervision may help improve surgical outcomes in resource-limited settings.

## Introduction

Unplanned surgical readmissions and returns to theater (RTTs) place a significant burden on both the healthcare system and patients undergoing surgery. The rate of unplanned RTTs in Iraq has not been previously reported in the literature. In Jordan, the rate of RTTs for gynecological procedures was reported to be 0.3% [1], while in the UK, it is 5.3% [2], highlighting the importance of monitoring and addressing these unexpected surgical outcomes.

Although there is a growing intent to improve surgical documentation and complication reporting, inconsistency remains in how surgical complications are reported. This underscores the need for a more consistent and structured reporting system to provide a reliable tool for appraising surgical techniques [3].

Emergency surgeries, such as laparotomies or obstetrical procedures, are associated with higher risks of hemorrhage, sepsis, and wound dehiscence [4,5]. In Iraq, where surgical care is under strain due to ongoing conflict, it is essential to understand the possible risk factors for RTTs to improve surgical outcomes [6]. Continuous staff training has been found to enhance the implementation of interventions and recognition of adverse effects, ultimately leading to better patient outcomes [7].

The World Health Organization (WHO) surgical safety checklist has been shown to reduce the rate of avoidable complications. The goal is to apply this checklist to all surgical patients [8]. In resource-limited settings, including some parts of the world, paper-based forms are still used, which can result in incomplete applications or even the loss of documentation. This, in turn, can increase the rate of preventable complications. Although the effectiveness of these interventions is well established globally, their implementation in low-resource settings remains underexplored.

This study evaluates the impact of a multimodal quality improvement program on RTT rates across two surgical specialties, general surgery and obstetrics and gynecology, at two tertiary hospitals in Iraq. One of the goals of clinical governance meetings is to reduce the incidence of complications, thereby avoiding unnecessary RTTs and prolonged hospital stays. Auditing these issues enhances our understanding of the problem and supports the development of structured frameworks for improved clinical practice.

The study specifically aimed to assess the impact of a multimodal quality improvement program, including staff training in surgical knot tying, adherence to the WHO surgical safety checklist, and consultant supervision, on RTT rates in general surgery and obstetrics and gynecology at two tertiary hospitals in Iraq.

## Materials and methods

Study design and setting

This two-center, quasi-experimental study was conducted at Al-Kindy Teaching Hospital (general surgery department) and Al-Elwyia Maternity Teaching Hospital (obstetrics and gynecology department) in Baghdad, Iraq. Data were collected over two cycles: the first cycle was a retrospective baseline, involving the collection of patient case files and morbidity and mortality (M&M) outcome reports for the period from January 2021 to January 2022; the second cycle was a prospective phase, which began after the implementation of the interventions, from March 2022 to March 2023.

Inclusion and exclusion criteria

All patients underwent abdominal laparotomy or laparoscopy in the general surgery center. During the study period, all cesarean sections, as well as laparotomies and laparoscopies for gynecological causes, were performed in the obstetrics and gynecology center. Cases in which the initial or subsequent operation occurred outside the study hospitals were excluded from the study (in-text citation).

Data collection

The following data were collected for all participants: age, sex, American Society of Anesthesiologists (ASA) class, urgency of the initial surgery, specialty (general surgery or obstetrics and gynecology), completion of the WHO safety checklist, whether a trainee or consultant performed the initial surgery, and, for those who RTT, the duration from the initial to the second procedure. Additional data included the time from a National Early Warning Score (NEWS) greater than one, which involves measuring vital signs along with oxygen requirements, pain score, and consciousness level, to the time of RTT, the reason for RTT, the assessment of preventability (based on M&M outcomes), and the impact on patient outcomes in terms of ICU admission, length of hospital stay, and the presence of a consultant during the second operation. These data were obtained from patient records. Cases with no documentation of the WHO checklist or with incomplete documentation were considered to have an incomplete checklist.

Classification of return to theater

RTTs were classified into three categories based on the outcomes of the M&M meetings. Preventable cases refer to those where RTT resulted from the omission of a critical step in management, the performance of an unnecessary step, or the use of an incorrect surgical technique. Examples include omitting antibiotics in an infected surgical site, performing a bowel anastomosis instead of creating a stoma in a severely inflamed bowel, or using a slippery knot (e.g., a granny knot) to secure a major bleeding site. Possibly preventable cases are those where improved preparation could have prevented RTT. For instance, performing a bowel anastomosis in malnourished patients without correcting the malnutrition beforehand or failing to reverse anticoagulants prior to surgery could lead to postoperative bleeding. Non-preventable cases represent those in which RTT occurred despite optimal care. An example includes pelvic collections following gynecological procedures or laparotomies, even after thorough washout and appropriate antibiotic use.

WHO safety checklist

The checklist used is a local modification of the original list [8]. Hospital documentation of the checklist was paper-based (see the Appendices section), and it was completed by the circulating nurse. The checklist was divided into three sections:

Sign-In (Conducted Before Starting Anaesthesia)

The nurse checks with the anesthesiologist to confirm the correct patient, procedure, site, consent, and marking of the procedure. They also verify that the anesthetic machine is functioning correctly, that anesthetic drugs are available and within their expiration dates, and that the pulse oximeter is connected. Drug allergies are noted and confirmed with the patient. The anesthesiologist also assesses the risk of a difficult airway and assigns the ASA grade.

Time-Out (Conducted Before Skin Incision)

The circulating nurse confirms the patient’s name, hospital number, and date of birth, as well as the name and site of the procedure, and the names and roles of the staff. They also confirm administration of antibiotics before skin incision (if indicated) and ask the surgeon whether any imaging needs to be displayed, the anticipated duration of the surgery, estimated blood loss, and any critical or unusual steps. Additional checks include patient warming, DVT prophylaxis, and protective measures to prevent patient positioning that could lead to falls. The scrub nurse is asked to confirm that the sterility indicators on the instruments have been checked and that there are no concerns with the instruments. Finally, both the surgeon and the anesthetist are asked about any patient-specific concerns.

Sign-Out (Conducted Before the Patient Leaves the Theater)

The procedure name is confirmed, specimen labels are verified, and instrument and sponge counts are confirmed to be correct. Any concerns regarding recovery are noted, and a further management plan is discussed.

Interventions

Based on the findings from the initial cycle, multi-modality interventions were applied:

Surgical Skill Training

The initial cycle showed that preventable RTTs due to bleeding were related to slipped ligatures. To address this, the educational center conducted workshops in the form of manikin-based ligation training, which lasted one day and was repeated three times at each center, covering all operating trainees. The training included instruction on different types of knots, the correct technique for performing hand-tied surgical knots and instrument knots, as well as one-on-one monitoring to ensure proper execution.

Adherence to the WHO Checklist

Another issue identified was suboptimal adherence to the WHO checklist. Following discussions with hospital managers, posters were displayed to highlight the steps in the checklist. Lectures were delivered on the importance of checklists and how to use them properly. Additionally, a "Checklist Guardian" role was assigned to the senior nurse in each operating theater to monitor compliance. The guardian was responsible for verbally reading each component of the checklist (provided in the appendix) and ensuring it was applied. At the end of the procedure, the guardian ensured that the complete checklist was documented and signed by the operating surgeon, anesthetist, and nurse in charge (the checklist guardian).

Supervision Policy

Some RTTs were linked to cases operated on solely by trainees without the presence of a consultant. After discussions with hospital management, it was decided to mandate the presence of consultants during high-risk procedures to supervise trainees. The supervision policy was enforced by the nurse in charge and the theater manager. High-risk patients were not brought to the theater unless they had an in-person discussion with the consultant surgeon, ensuring adequate supervision during the procedure.

Statistical analysis

Data collection was based on a paper-based form that included the required variables. The data were then transferred to SPSS Statistics version 26 (IBM Corp. Released 2019. IBM SPSS Statistics for Windows, Version 26.0. Armonk, NY: IBM Corp.) for statistical analysis. The distribution of continuous variables (age, duration from the initial procedure to the second procedure, and hospital stay duration) was tested using the Shapiro-Wilk test, which indicated that all variables were skewed. Therefore, Mann-Whitney U tests were used. Categorical data were coded in numerical form for ease of analysis, and statistical analysis was performed using chi-square or Fisher’s exact tests. Statistical significance was set at p < 0.05.

Ethical considerations

Approval was obtained from the scientific and ethical councils of both hospitals: Protocol 3221A on December 12, 2021, at Al-Kindy Teaching Hospital, and Protocol 1224 on December 15, 2021, at Al-Elwyia Maternity Teaching Hospital. Patient consent for the first cycle was waived due to the study's retrospective nature, while written consent was obtained from patients included in the second cycle. The study was conducted according to the Declaration of Helsinki.

## Results

A total of 1,133 surgeries were included (670 in the first cycle and 463 in the second). An unplanned RTT occurred in 70 cases overall (6.2%). The RTT incidence decreased significantly between cycles, from 7.9% in the first cycle to 3.7% in the second (p = 0.004). This reduction was observed across specialties: the RTT rate in general surgery declined from 6.3% to 3.0% (p = 0.048), and in obstetrics and gynecology from 10.5% to 4.8% (p = 0.037) (Table 1).

**Table 1:** Distribution of RTT cases according to cycle and specialty RTT: return to theater

RTT	First cycle n=670	Second cycle n=463	Total n=1133	p-value
	No. (%)	No. (%)	No. (%)	
Overall				
Yes	53 (7.9)	17 (3.7)	70 (6.2)	0.004
No	617 (92.1)	446 (96.3)	1063 (93.8)	
General surgery				
Yes	26 (6.3)	9 (3)	35 (4.9)	0.048
No	388 (93.7)	288 (97)	676 (95.1)	
Total	414 (100)	297 (100)	711 (100)	
Obstetrics and gynecology				
Yes	27 (10.5)	8 (4.8)	35 (8.3)	0.037
No	229 (89.5)	158 (95.2)	387 (91.7)	
Total	256 (100)	166 (100)	422 (100)	

Patients who required reoperation differed from those who did not in several key aspects. Urgent (non-elective) initial surgeries were associated with a higher risk of RTT compared to elective surgeries. Incomplete documentation of the WHO surgical safety checklist was observed more frequently in RTT cases. The median postoperative hospital stay was significantly longer for the RTT cohort (24 vs. 9 days, p < 0.001). Patient age, sex, ASA physical status, and the seniority of the primary surgeon did not differ significantly between patients with and without RTT (Table 2).

**Table 2:** Analysis of risk factors for RTT in all cases regardless the cycle * Data presented as median (minimum-maximum) ASA: American Society of Anesthesiologists, WHO: World Health Organization

RTT					
Variables		Yes	No	Total	p-value
		No. (%)	No. (%)	No. (%)	
Age (years)*		34 (17-72)	36 (16-73)	35 (16-73)	0.736
Sex	Male	22 (31.4)	308 (29)	330 (29.1)	0.662
	Female	48 (68.6)	755 (71)	803 (70.9)	
ASA grade	I	38 (54.3)	650 (61.1)	688 (60.7)	0.148
	II	21 (30)	215 (20.2)	236 (20.8)	
	III	11 (15.7)	198 (18.6)	209 (18.4)	
	IV	0 (0)	0 (0)	0 (0)	
Urgency	Urgent	54 (77.1)	515 (48.4)	569 (50.2)	<0.001
	Elective	16 (22.9)	548 (51.6)	564 (49.8)	
Consultant/trainee	Consultant	57 (81.4)	839 (78.9)	896 (79.1)	0.618
	Trainee	13 (18.6)	224 (21.1)	237 (20.9)	
WHO checklist filled	Complete	26 (37.1)	896 (84.3)	922 (81.4)	<0.001
	Incomplete	44 (62.9)	167 (15.7)	211 (18.6)	
Hospital stay (days)*		24 (14-31)	9 (1-17)	10 (1-31)	<0.001

Among the 70 cases with unplanned reoperation, sepsis was the most frequent indication (40.0%, 28/70), followed by wound dehiscence (34.3%, 24/70) and postoperative hemorrhage (25.7%, 16/70). A consultant surgeon was present for 92.9% of these reoperations, indicating a high level of senior involvement. The median interval from the initial operation to RTT was similar in the first and second cycles (7 days vs. 5 days, p = 0.14). Likewise, the proportion of patients requiring postoperative admission to the intensive care unit after reoperation did not change significantly between cycles (p = 0.703). However, the duration from a NEWS score greater than 1 to the second surgery was shorter in the second cycle (Table 3).

**Table 3:** Analysis of RTT cases according to study cycle * Data presented as median (minimum-maximum) ICU: intensive care unit, NEWS: National Early Warning Score

Variables		First cycle	Second cycle	Total	p-value
		No. (%)	No. (%)	No. (%)	
Reason	Bleeding	14 (26.4)	4 (23.5)	18 (25.7)	0.146
	Sepsis	18 (34)	10 (58.8)	28 (40)	
	Wound dehiscence	21 (39.6)	3 (17.6)	24 (34.3)	
Preventability assessment	Preventable	4 (7.5)	2 (11.8)	6 (8.5)	0.84
	Possibly preventable	18 (34)	5 (29.4)	23 (32.9)	
	Non-preventable	31 (58.5)	10 (58.8)	41 (58.6)	
Presence of a senior in the second operation	Yes	49 (92.5)	16 (94.1)	65 (92.9)	0.817
	No	4 (7.5)	1 (5.9)	5 (7.1)	
ICU admission	Yes	132 (19.7)	87 (18.8)	51 (72.9)	0.703
	No	538 (80.3)	376 (81.2)	19 (27.1)	
Time from initial to second surgery (days)*		7 (2-15)	5 (1-10)	6 (1-15)	0.14
Duration from NEWS 1 to theater (hours)*		31 (15-48)	23 (11-37)	28.5 (11-48)	0.003
Hospital stay (days)*		10 (1-31)	9 (1-28)	24 (14-31)	0.171

Each RTT case was reviewed for preventability using outcomes from multidisciplinary M&M meetings. Of the 70 cases, 8.5% (6/70) were classified as preventable, 32.9% (23/70) as possibly preventable, and 58.6% as non-preventable. Preventability often aligned with the underlying cause. In the first cycle, one case of bleeding was considered preventable and was attributed to an insecure surgical knot placed by a trainee; a slipped ligature was identified during reoperation. One sepsis-related case in the first cycle and two cases in the second cycle were classified as preventable, where no antibiotics were administered despite a perforated viscus. Two wound dehiscence cases were also deemed preventable due to the wide spacing between sutures at the initial procedure. Cases considered possibly preventable included those where the consultant was not present during surgery or where the WHO surgical safety checklist was not fully completed. Wound dehiscence was frequently assessed as possibly preventable, while most sepsis cases, especially in the second cycle, were judged non-preventable due to the inherent severity of the primary pathology (Table 4).

**Table 4:** Analysis of the second operation causes and the preventability RTT: return to theater

Reason for RTT	Preventability assessment			
	Preventable	Possibly preventable	Non-preventable	Total
	No. (%)	No. (%)	No. (%)	No. (%)
First cycle				
	n=4	n=18	n=31	n=53
Bleeding	1 (25)	8 (44.4)	5 (16.1)	14 (26.4)
Sepsis	1 (25)	3 (16.7)	14 (45.2)	18 (34)
Wound dehiscence	2 (50)	7 (38.9)	12 (38.7)	21 (39.6)
Second cycle				
	n=2	n=5	n=10	n=17
Bleeding	0 (0)	2 (40)	2 (20)	4 (23.5)
Sepsis	2 (100)	0 (0)	8 (80)	10 (58.8)
Wound dehiscence	0 (0)	3 (60)	0 (0)	3 (17.6)

## Discussion

This study investigated the application of multimodal quality improvement interventions and found that they significantly reduced unplanned RTT rates in both general surgery and obstetrics and gynecology at two tertiary hospitals in Iraq. The overall RTT rate declined from 7.9% to 3.7%, with reductions observed in both specialties. This highlights the potential impact of structured interventions in resource-limited settings. The use of multimodality interventions synergistically improved RTT rates by addressing various challenges in surgical care. This reduction in RTT contributes to improved patient quality of life and further decreases the costs associated with secondary operations.

Our findings align with existing evidence suggesting that RTTs are a sensitive indicator of surgical quality. A recent UK-based study reported a similar RTT rate of 5.3% in emergency general surgery, identifying hemorrhage and inadequate drainage as leading causes, many of which were potentially preventable through improved technique and perioperative planning [2]. In our cohort, sepsis (40%), wound dehiscence (34.3%), and hemorrhage (25.7%) were the predominant causes of reoperation, mirroring global trends in surgical complications.

The decline in RTT observed in our second cycle may be attributed to several specific interventions. First, training workshops focused on secure surgical knot tying directly addressed preventable bleeding complications noted in the initial cycle. The importance of surgical technique in preventing RTT is well-documented. A retrospective cohort study found that technical errors, including inadequate hemostasis, accounted for up to 48% of reoperations in orthopedic settings [9].

Second, improved compliance with the WHO surgical safety checklist proved critical. Our study found significantly lower checklist completion in RTT cases, a trend echoed in global literature. A quality improvement initiative that integrated surgical safety protocols with team-based discussions led to improved adherence and was associated with reductions in critical care admissions and improved preoperative preparation [10]. Furthermore, assigning checklist guardians, such as senior nurses, is a low-cost strategy that has been shown to enhance compliance and team accountability [11].

Our data also suggest that consultant supervision during high-risk operations may help prevent RTT. Although overall consultant involvement did not statistically differ between RTT and non-RTT cases, qualitative review identified consultant absence as a contributing factor in some preventable reoperations. Similar findings were reported in a review of gynecological reoperations, where surgeries led by consultants were associated with lower complication and reoperation rates [1].

Notably, while our study observed improvements in RTT rates and checklist adherence, specific outcomes, such as ICU admission and time from initial surgery to reoperation, remained unchanged. This may reflect the inherent severity of emergency cases, particularly those involving sepsis, which were frequently deemed non-preventable. These findings are consistent with broader surgical literature, where RTT rates must be interpreted in context. A higher RTT rate does not always indicate poor care and may instead reflect timely interventions for evolving complications [12].

Overall, our study demonstrates that even in resource-constrained environments, practical, team-based interventions, such as staff education, surgical supervision, and protocol adherence, can meaningfully reduce preventable surgical complications and RTT rates.

Study limitations

The data collection in the initial cycle was retrospective, which presents the limitation of possible loss of the WHO checklist (especially as it was paper-based), potentially leading to a higher rate of incomplete documentation. The study lacked a control group. Although the current study included pre- and post-intervention data collection, it did not have a parallel comparison with a control group. This was due to the impracticality of training only part of the staff while leaving others untrained, or enforcing the application of the WHO checklist and consultant supervision for one group but not the other. The use of multiple interventions showed a significant improvement in the rate of RTT; however, the effect of individual interventions could not be measured. The study involved two centers, which provided a good sample size; however, including more centers could have yielded more generalizable outcomes.

## Conclusions

Unplanned RTT in general surgery and obstetrics and gynecology is a significant issue, often linked to preventable factors such as poor surgical technique, incomplete safety checklists, and a lack of senior supervision. A targeted, multi-modal quality improvement program that focused on staff training, checklist enforcement, and the presence of consultants during high-risk operations led to a meaningful reduction in reoperation rates in resource-limited settings. This study provides insight into the current limitations in low-resource settings and could guide future improvements in patient care. Future studies, including those involving more centers and a longer follow-up period, will provide more generalizable results.

## Figures and Tables

**Figure 1:**
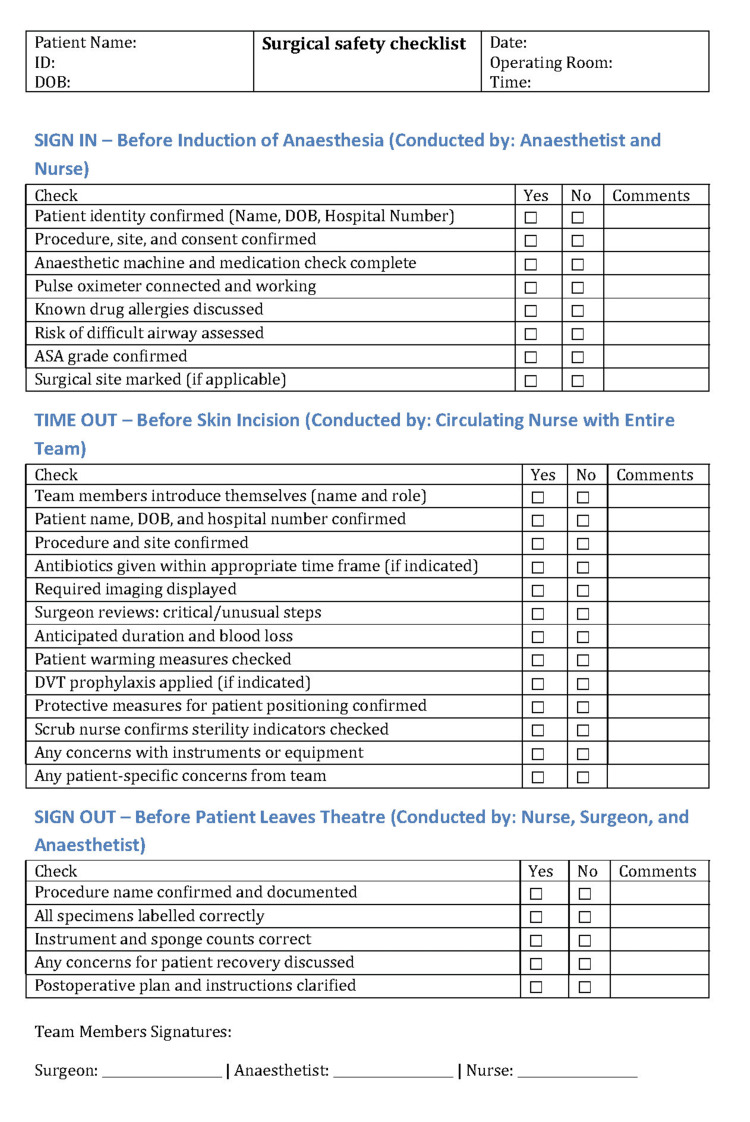
Modified WHO safety checklist used in the study centers Sample of the WHO safety checklist used in the study centers, based on the original WHO checklist with local modifications. WHO: World Health Organization, ID: identification number, DOB: date of birth, ASA: American Society of Anesthesiologists, DVT: deep vein thrombosis [8]
